# Vitrectomy with or without internal limiting membrane peeling for myopic foveoschisis

**DOI:** 10.1186/s12886-020-01354-8

**Published:** 2020-03-04

**Authors:** Junmin Gui, Ling Ai, Ting Huang

**Affiliations:** Department of Retinal & Vitreous Diseases, Chongqing Aier-Mega Eye Hospital, Aier Medical Group, 288 Nanchen Street, Chongqing, 400060 People’s Republic of China

**Keywords:** Myopic foveoschisis, Internal limiting membrane, Vitrectomy, Indocyanine green

## Abstract

**Background:**

The aim of this study was to compare the anatomical and visual outcomes of vitrectomy with or without internal limiting membrane (ILM) peeling for symptomatic myopic foveoschisis (MF).

**Methods:**

A retrospective cohort study of patients who had undergone vitrectomy for symptomatic MF at our specialist ophthalmology department in China. Cases were retrospectively categorized into one of two cohorts, depending on whether or not they had undergone ILM peeling (ILMP) during their surgery. Over a mean follow-up period of 18 months, all eyes underwent repeated examinations, including best-corrected visual acuity (BCVA) and optical coherence tomographic (OCT) recordings, particularly focusing on central foveal thickness (CFT), macular hole (MH) formation and/or foveal detachment (FD).

**Results:**

We included 32 eyes (32 patients) with mean age of 62.2 ± 7.4 years. 31 patients (96.8%) were female. There were 21 eyes in the ILMP cohort and 11 eyes in the non-ILMP cohort. There were no significant preoperative differences in age, axial length, symptom duration or postoperative follow-up period between the two cohorts. MF was resolved completely in all of the eyes except one eye in the ILMP cohort. The postoperative CFT was significantly reduced compared to the preoperative baseline in both cohorts (469 ± 203 μm to 253 ± 56 μm; *p* = 0.003 in no-ILMP; 495 ± 178 μm to 244 ± 63 μm; *p* <  0.001 in ILMP, respectively). The final BCVA improved significantly in non-ILMP (1.27 ± 0.63 logMAR to 0.73 ± 0.55 logMAR; *p* = 0.021); but not significantly in ILMP cohort (1.25 ± 0.51 to 0.98 ± 0.57 logMAR; *p* = 0.143).

**Conclusion:**

Vitrectomy, either with or without ILM peeling, results in a significant anatomical improvement in eyes with MF. Eyes treated by vitrectomy may have a better visual improvement when ILM was not peeled.

## Background

Myopic foveoschisis (MF) was first described by Takano and Kishi in 1999, and is recognized as one of the major complications associated with pathological myopia [1, 2]. Many subsequent studies have demonstrated that vitrectomy with internal limiting membrane peeling (ILMP) can be effective in producing both anatomical and functional improvement in MF [3–7]. In order to reduce postoperative macular hole (MH) formation and foveal damage, a partial ILMP technique with foveal sparing was proposed [8], but there is little evidence that this technique has better outcomes than complete ILMP [9, 10] Furthermore, more recent studies have shown similar anatomical and functional improvements can be achieved without any form of IMLP [4, 11], casting doubt on the benefits of ILMP.

In this study we retrospectively analyze the medical records of patients with MF who were treated by vitrectomy at our specialist ophthalmology department in China. Specifically, we divide the patients retrospectively into two cohorts — those who underwent ILMP and those who didn’t — and compare the two.

## Methods

Ethics panel approval for this study was obtained from the Medical Ethics Committee of Chongqing Aier-Mega Eye Hospital. All methods were carried out in accordance with the tenets of the Declaration of Helsinki. We retrospectively reviewed the medical records of patients who underwent vitrectomy for MF between January 2014 and April 2018 at the Chongqing Aier-Mega Eye Hospital in Chongqing, China.

Thirty-two eyes from 32 patients with symptomatic MF were included. All the included patients had progressive visual deterioration and/or metamorphopsia, that was attributable to MF. Eyes with macular holes were not excluded unless there was associated retinal detachment clearly visible on B-ultrasound examination. All included patients had undergone surgical vitrectomy (following written informed consent) as a treatment for MF. All surgeries were performed by one experienced surgeon (J.G.). There was no prospective (preoperative) allocation of patients to groups as part of this study. The decision whether or not to perform ILMP during surgery was at the discretion of the surgical team and varied depending on the particular patient. For this study, patients were retrospectively assigned to one of two cohorts — ILMP and non-ILMP — on the basis of whether or not they had ILMP during surgery.

As part of their routine work-up, all patients had a complete ophthalmic examination, including measurement of best-corrected visual acuity (BCVA), refraction, axial length (AXL), slit-lamp examination, fundus examination and optical coherence tomography (OCT). Preoperative and postoperative BCVAs were determined as decimal visual acuity and then converted to logarithm of the minimum angle of resolution (logMAR) units for statistical analysis. We also defined an improvement or worsening in postoperative BCVA as a change of 0.2 logMAR units from baseline.

### Surgical procedures

Patients underwent a standard three-port 25-gauge sutureless pars plana vitrectomy under retrobulbar anesthesia. Phacoemulsification with intraocular lens implantation was performed in 26 eyes. After core vitrectomy, triamcinolone acetonide was injected to identify, and help remove, any residual posterior vitreous. In patients who underwent ILMP, the ILM was stained with 0.25% indocyanine green (ICG) and peeled up to the superior and inferior vascular arch using intraocular forceps. Air-fluid exchange was done in all the eyes. At the completion of surgery, eyes were filled with 15% perfluoropropane in 19 eyes, air in 3 eyes and silicone oil in 10 eyes (those with full thickness macular holes with foveal detachment). Patients were encouraged to maintain a prone posture for at least a week postoperatively. Silicone oil was removed in all 10 eyes between 3 and 8 months postoperatively.

### Statistical analysis

Statistical analysis was performed in SPSS Statistics (version 21; IBM Corporation, Armonk NY, USA). Numerical variables were analyzed using Mann-Whitney U-tests and Wilcoxon paired signed-rank tests (because the data did not meet parametric assumptions). Qualitative variables are expressed as frequencies and percentages and were analyzed using chi-square test or Kruskal-Wallis test. Associations between the final BCVA and the clinical parameters were examined by univariate analysis. Differences were considered significant when the probability of Type 1 error (*p*-value) was < 0.05.

## Results

### Preoperative characteristics

Thirty-two eyes of 32 consecutive patients (31 females, 96.8%) were retrospectively included in the study. Their mean age was 62.2 ± 7.4 (range: 48–75 years) ; the mean axial length was 29.0 mm (range: 26.2–33.0 mm) and their mean symptom duration was 10.5 months (range: 0.5–24 months) (Table 1). Thirty eyes were phakic and 2 were pseudo phakic. All patients underwent vitrectomy, but 21/32 (66%) had concomitant ILMP and 11/32 (34%) did not have ILMP. Patients were followed up for 6 to 55 months with a mean period of 18.0 months and retrospectively divided into two cohorts based on their exposure to ILMP during vitrectomy. Preoperatively, there were no significant differences in age, axial length, symptom duration and the proportion of FD or MH between the two cohorts and there was no statistical difference in the proportion of undergoing phaco during vitrectomy between the two groups, neither. (Table 1).

**Table 1:** Clinical characteristics of patients (pre-operative)

Parameter	Cohort (retrospectively grouped)		***P***-value^*^
	No ILMP (*n* = 11)	ILMP (*n* = 21)	
**Age (years)**	63.2 ± 7.4	61.8 ± 7.5	0.676
**Symptom duration(months)**	**10.9 ± 10.5**	**10.3 ± 9.7**	**0.642**
**Axial length (mm)**	**29.2 ± 2.0**	**28.9 ± 2.1**	**0.706**
**Refractive error (Diopter)**	−13.1 ± 8.2	−11.1 ± 4.9	0.388
**Foveal detachment present**	4/11 (36.3%)	11/21 (52.3%)	0.472
**Macular hole present**	4/11 (36.4%)	4/21 (19.0%)	0.397
**Phaco performed during vitrectomy**	8/11(72.7%)	18/21(85.7%)	0.390
**Follow-up (months)**	16.6 ± 11.2	18.7 ± 13.9	0.658

Continuous variables are given as mean ± standard deviation (SD). Binary variables are given as fraction (percentage in brackets)

^*^P-value of there being a difference between the no-ILMP and ILMP cohort (Mann–Whitney U-test for continuous variables and Chi-square test and Fisher’s exact test for binary variables)

### Surgical outcomes

**Fig. 1:**
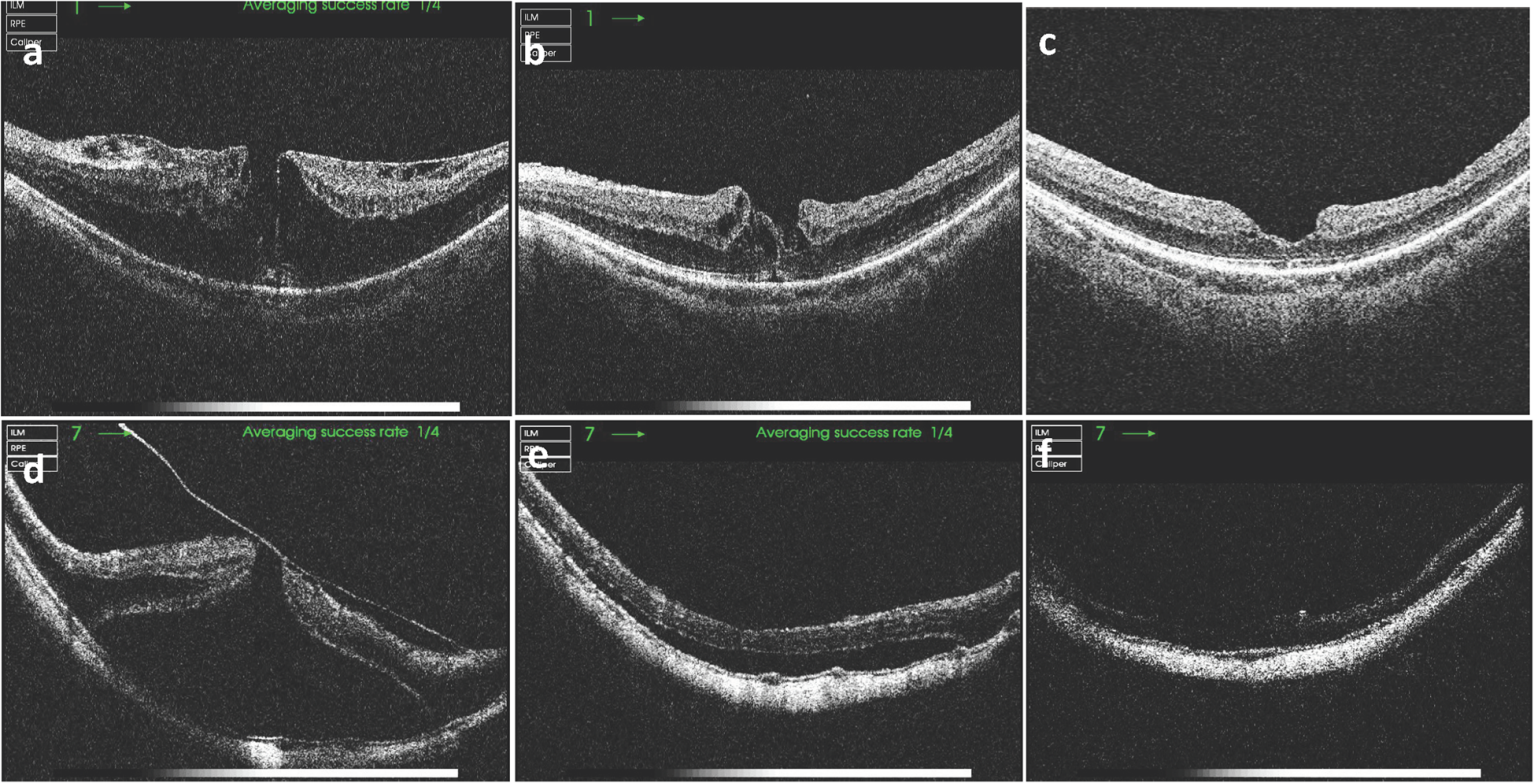
OCT imaging of myopic foveoschisis (MF) before and after vitrectomy without ILM peeling. (**a**) Preoperative image of the eye of a 72-year-old female with MF; AXL 26.1 mm and BCVA 0.2. (**b**) Two months postoperatively, MF partially resolved. (**c**) 40 months postoperatively, MF completely resolved; BCVA improved to 0.6. (**d**) Preoperative image of the eye of a 51-year-old female with MF, a macular hole and foveal detachment; AXL 32.2 mm, BCVA 0.05. (**e**) Two months postoperatively, foveal detachment partially resolved. (f) 19 months postoperatively, MF completely resolved; BCVA improved to 0.2 OCT, optical coherence tomography; AXL, axial length; BCVA, best corrected visual acuity.

**Fig. 2:**
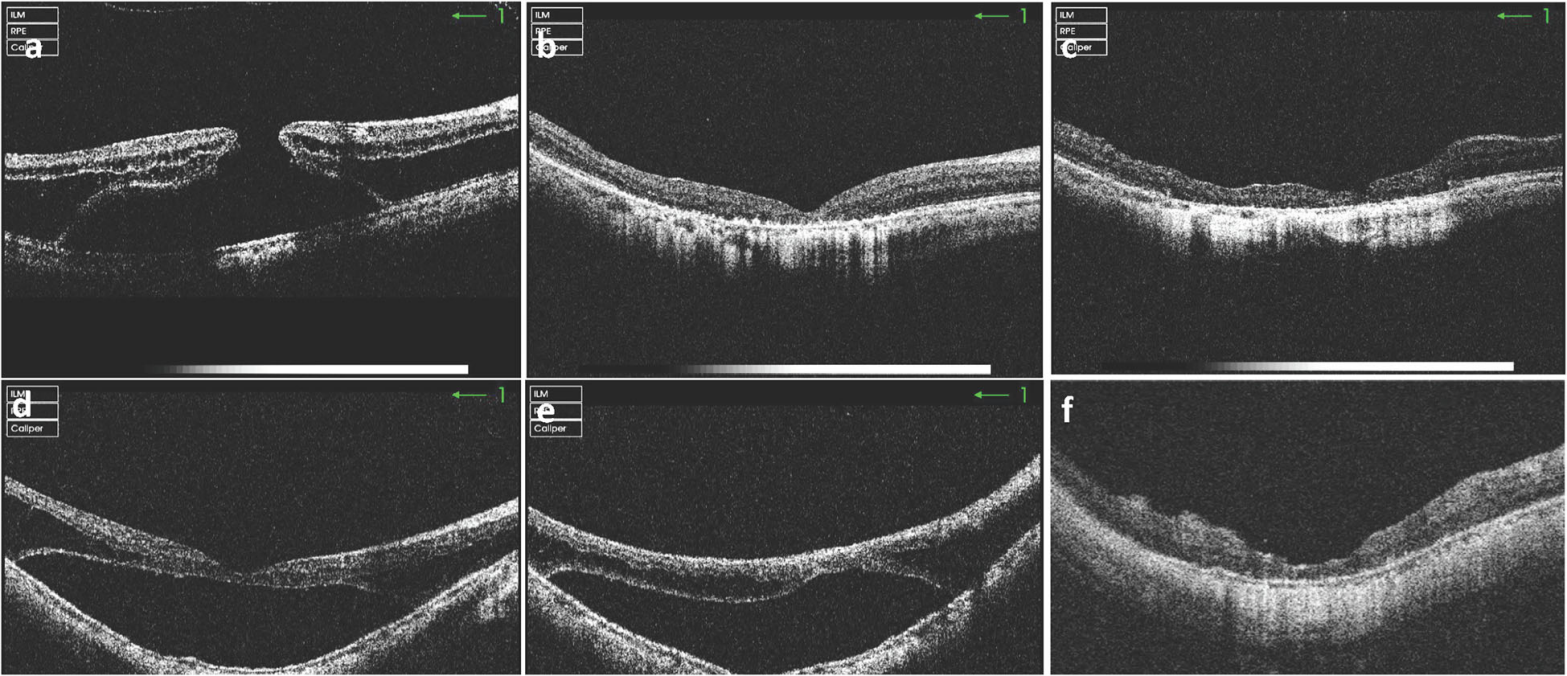
OCT imaging of myopic foveoschisis (MF) before and after vitrectomy with ILM peeling. (**a**) Preoperative image of the eye of a 51-year-old female with MF, a macular hole and foveal detachment; AXL 26.0 mm and BCVA CF/50 cm. (**b**) Two months postoperatively, the macular hole was closed. (**c**) Twelve months postoperatively, the foveal contour was stable; BCVA remained unchanged. (**d**) Preoperative image of the eye of a 64-year-old female with foveal detachment; AXL 26.3 mm and BCVA CF/15 cm. (**e**) Two month postoperatively, foveal detachment unchanged. (**f**) Nine months postoperatively, schisis and foveal detachment resolved completely; BCVA improved to 0.12. OCT, optical coherence tomography; AXL, axial length; BCVA, best corrected visual acuity; CF, counting finger

MF resolved completely in all patients following surgery, except one in the ILMP cohort. Central foveal thickness was significantly reduced compared to preoperative values in both cohorts (469 ± 203 μm to 253 ± 56 μm; *p* = 0.003 in no-ILMP; 495 ± 178 μm to 244 ± 63 μm; *p* <  0.001 in ILMP), but there was no difference between the two cohorts (Table 2). MF usually resolved within the first few postoperative months, but in some cases, it took longer to achieve complete resolution (Figs. 1 and 2).

**Table 2:** Anatomical and visual outcomes of vitrectomy

Parameter	Cohort (retrospectively grouped)		***P***-value^*^
	No ILMP (*n* = 11)	ILMP (*n* = 21)	
**BCVA (logMAR)**			
Preoperative	1.27 ± 0.63	1.25 ± 0.51	0.904*
Postoperative (final)	0.73 ± 0.55	0.98 ± 0.57	0.340*
*P*-value†	0.012	0.143	–
**Categorical change in BCVAǂ**			0.072§
Improved	10 (90.9%)	12 (57.1%)	–
Unchanged	0 (0.0%)	4 (19.1%)	*–*
Worsened	1 (9.1%)	5 (23.8%)	–
**Central foveal thickness (μm)**			
Preoperative	469 ± 203	495 ± 178	0.592*
Postoperative	253 ± 56	244 ± 63	0.111*
*P*-value†	0.003	< 0.001	–
**Postoperative complications**			
Full thickness macular hole	0	1 (5.6%)	*–*
Retinal detachment	0	2 (11.1%)	*–*

Numerical variables are given as mean ± standard deviation (SD). Categorical variables are given as count (percentage in brackets)

^*^, *P*-value between no-ILMP and ILMP cohort (Mann-Whitney U-test). †, *p*-value between preoperative and postoperative values (Wilcoxon paired signed-rank test). ‡ An improvement or worsening of visual acuity was defined as a change 0.2 logMAR units. §, *p*-value between Non-peel and ILM peel group (Kruskal–Wallis test)

The final visual acuity improved significantly in no-ILMP group, from 1.27 ± 0.63 logMAR preoperatively to 0.73 ± 0.55 logMAR postoperatively (*p* = 0.021; Table 2); BCVA was also improved in the ILMP cohort (1.25 ± 0.51 logMAR to 0.98 ± 0.57 logMAR), but this improvement was not statistically significant (*p* = 0.143; Table 2). The proportion of eyes with meaningful improvement (defined as 0.2 logMAR units) in BCVA was not significantly different between the two cohorts (*p* = 0.072; Table 2). Final BCVA in the no-ILMP cohort was improved in 90.9% of patients and was worse than preoperative in 9.1% of eyes. Final BCVA in the ILMP cohort was improved in 57.1%, unchanged in 19.1% and was worse in 23.8% of eyes.

Combining data from both cohorts, a significant correlation was found between final BCVA and preoperative BCVA (Pearson’s correlation coefficient, r = 0.410; *p* = 0.003), but no significant correlations were found between final BCVA and age (*p* = 0.493), axial length (*p* = 0.824), symptom duration(*p = 0.051*), preoperative refractive error (*p* = 0.125) or preoperative central foveal thickness (*p* = 0.383). There was no correlation between the improvement of BCVA and the presence of a FD (*p* = 0.23) or presence of MH (*p* = 0.83).

### Surgical complications

Two eyes in the ILMP group developed retinal detachment postoperatively. One of these was because of postoperative macular hole formation. The MF in this case was partially resolved initially but worsened gradually. A year later, a foveal detachment occurred, which soon after developed into a full-thickness macular hole, followed by retinal detachment. This patient was successfully treated with retinal reattachment and closure of the macular hole. Another case had a posterior retinal detachment with a tiny hole, detected by OCT, at the nasal rim of optic disc. This case was also successfully repaired by re-operation.

## Discussion

Our results demonstrate that, for the treatment of myopic foveoschisis (MF), vitrectomy without internal limiting membrane peeling (ILMP) has similar outcomes to vitrectomy with ILMP. This is consistent with previous reports [4, 11]. Indeed, within-cohort statistical analysis showed that significant visual improvement was only achieved in the no-ILMP cohort. The percentage of eyes with a significant improvement of the final BCVA was also higher in the no-ILMP cohort (90.9%) than that in the ILMP cohort (57.1%), although this difference was not statistically significant (*p* = 0.072). A direct between-cohort statistical comparison of BCVA improvement did not demonstrate a significant advantage of vitrectomy without ILMP, although this may be a false negative, due to the lower power of the unpaired test. A larger study, ideally prospective, is required to support a definitive conclusion on the advantage of vitrectomy without ILMP.

As MF mainly occurs in patients with pathological myopia (especially those with staphylomas), ILMP in such patients is often difficult and ILM staining is necessary. Due to its availability, indocyanine green (ICG) is widely used (including by us) to stain the ILM. Although some studies demonstrate that ICG-assisted vitrectomy combined with ILMP is safe and effective in the treatment of idiopathic macular holes and epiretinal membranes [12, 13], animal studies show that 0.05% ICG can cause neuroretinal thinning in rats and reduced mfERG responses in pigs [14, 15]. Using multifocal electroretinography, Lai and colleagues demonstrate functional changes in the macula during epiretinal membrane surgery with ICG-assisted ILMP, and show that higher concentrations of ICG (1.25 mg/ml) produce significant reductions in N1 and P1 response amplitudes compared with baseline [16]. Furthermore, Sayanagi and colleagues show that the pattern of ICG residue in severe myopia is different from those in non-myopics [17]. In eyes with patch atrophy, ILMP is so difficult that repeated staining is often required, increasing the potential retinal toxicity of ICG. We therefore suggest that ICG toxicity may (at least partly) explain why performing ILMP with vitrectomy does not improve postoperative visual acuity as much as might be expected.

There is a similar debate on the benefits and harms of ILMP in the treatment of epi-macular membranes. Opponents of ILMP show that the visual outcomes between eyes that undergo ILMP and those that do not are the same. Stripping of the ILM removes the Müller cell footplates, so it is reasonable to expect some Müller cell dysfunction. Adverse events such as eccentric para-central macular holes, macular microscotomata, and retinal dimpling have also been associated with ILMP [18]. Such adverse events may be even more common in pathological myopia, characterized by long axial length and diffuse chorioretinal atrophy. To prevent postoperative macular hole formation and possible damage to the fovea, a fovea-sparing ILMP technique has been advocated [8, 19, 20], but some studies have shown that there is no difference in postoperative visual acuity between fovea-sparing ILMP and complete ILMP [9, 10].

In the current study, two eyes in the ILMP cohort developed postoperative retinal detachment. One was associated with postoperative macular hole formation, as a result of the progression of unresolved MF. The other was caused by a posterior hole unrelated to ILMP, but which may have been caused by the surgical separation of the remaining posterior vitreous. This highlights the risks of performing suction or peeling on the residue of the posterior vitreous.

There are many limitations to our current study. It is a retrospective, non-randomized cohort study with a small number of cases. Although the average follow-up time is long enough, it may not be sufficient for some cases. We did not find a significant difference in visual acuity improvement between ILMP and no-ILMP groups, but it is possible that this was a false negative, due to the limited sample size available for this study. Our data suggest that vitrectomy without ILMP may indeed produce better outcomes than vitrectomy with ILMP, but ideally a prospective, randomized-controlled trial is required (with appropriate pre-trial power calculations and sufficient recruitment) to assess which approach is better. The same (or a separate) study should also be designed to assess the benefits and harms of ICG dye.

In conclusion, we show that vitrectomy without ILMP for MF has comparable anatomical outcomes to vitrectomy with ILMP. We also provide some evidence that visual outcomes are better without ILMP, and we highlight the potential retinal toxicity of the ICG dye commonly used in ILMP.

## Data Availability

The datasets used and/or analyzed during the current study are available from the corresponding author on reasonable request.

## Abbreviations

BCVA: Best corrected visual acuity
FD: Foveal detachment
ICG: Indocyanine green
ILM: Internal limiting membrane
logMAR: Logarithm of the minimum angle of resolution
MF: Myopic foveoschisis
MH: Macular hole
OCT: Optical coherence tomography
